# Improved Conventional and New Approaches in the Diagnosis of Tuberculosis

**DOI:** 10.3389/fmicb.2022.924410

**Published:** 2022-05-31

**Authors:** Baoyu Dong, Zhiqun He, Yuqing Li, Xinyue Xu, Chuan Wang, Jumei Zeng

**Affiliations:** ^1^West China School of Public Health and West China Fourth Hospital, Sichuan University, Chengdu, China; ^2^State Key Laboratory of Oral Diseases, National Clinical Research Center for Oral Diseases, West China Hospital of Stomatology, Sichuan University, Chengdu, China

**Keywords:** tuberculosis, *Mycobacterium tuberculosis*, early detection, diagnostic approaches, improved conventional methods

## Abstract

Tuberculosis (TB) is a life-threatening infectious disease caused by *Mycobacterium tuberculosis (M. tuberculosis)*. Timely diagnosis and effective treatment are essential in the control of TB. Conventional smear microscopy still has low sensitivity and is unable to reveal the drug resistance of this bacterium. The traditional culture-based diagnosis is time-consuming, since usually the results are available after 3–4 weeks. Molecular biology methods fail to differentiate live from dead *M. tuberculosis*, while diagnostic immunology methods fail to distinguish active from latent TB. In view of these limitations of the existing detection techniques, in addition to the continuous emergence of multidrug-resistant and extensively drug-resistant TB, in recent years there has been an increase in the demand for simple, rapid, accurate and economical point-of-care approaches. This review describes the development, evaluation, and implementation of conventional diagnostic methods for TB and the rapid new approaches for the detection of *M. tuberculosis*.

## Introduction

**Figure 1:**
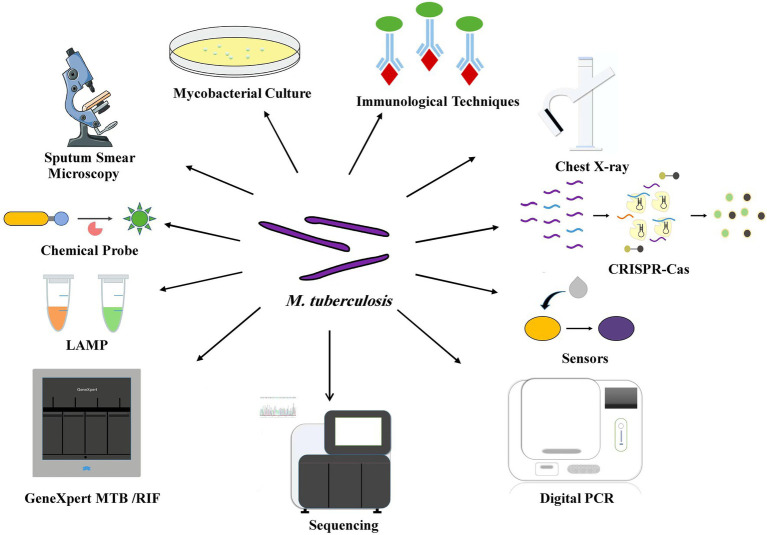
Various diagnostic tools for TB point-of-care testing.

Tuberculosis (TB) is a chronic infectious disease caused by *Mycobacterium tuberculosis*. Delayed TB diagnosis causes the infected individuals to act as a reservoir for *M. tuberculosis* with the potential to infect other individuals. Early and rapid diagnosis of TB is essential to improve the efficacy of the treatment and effectively block the interpersonal transmission. However, many suspected TB patients cannot be diagnosed immediately, especially when patients face a variety of problems, such as the economic burden of transportation, which further exacerbates the lack of diagnosis (Lönnroth et al., 2010). The gap between traditional diagnostic methods for TB and the actual clinical needs requires the development of new diagnostic methods that should be accurate, rapid and cost-effective. This review not only incorporates the improvement and optimization of conventional methods, but also describes the creation and development of new diagnostic approaches (Figure 1).

## Improvement and Optimization of Current Diagnostic Techniques

### Etiological Diagnostic Techniques

#### Sputum Smear Microscopy

SSM is the preferred method in the diagnosis of pulmonary TB. SSM is actually the only approach for TB diagnosis in some remote areas of developing countries (Ben-Selma et al., 2009). The commonly used staining methods are Ziehl-Neelsen (ZN) staining and fluorescent staining (Auramine-O / Auramine-rhodamine). SSM is easy to perform and cost-effective, but it lacks sensitivity (>10^4^ bacilli•mL-1 of sputum to get positive) and has a high false negative rate, which is prone to cause misdiagnosis. In recent years, the operator-independent SSM based on the ZEISS Axio Scan has been developed and evaluated to automatically detect and count acid-fast bacilli with a sensitivity of 97.06% and a specificity of 86.44% (Zingue et al., 2018). This method improves the detection efficiency and saves valuable time for the laboratory staff. The specific fluorescent antibody labeling with laser confocal microscopy has been developed to improve the efficiency of *M. tuberculosis* detection in lung tissue samples, especially when these bacteria have weak ZN staining (Erokhina et al., 2019). The Pat-Scan program constructed using digital pathology has been developed to detect and quantify the bacteria in the paraffin-embedded ZN-stained tissue, thus being helpful in reducing the diagnostic time (Sua et al., 2021). It is worth noting that SSM can neither distinguish dead and live bacteria, nor distinguish *M. tuberculosis* from nontuberculosis mycobacteria. However, ZN staining can provide information on the acid-fastness of bacilli existing in the patients samples. This information is sometimes critical in the samples of the patients who had already been treated with anti-TB drugs. Furthermore, this information can be obtained from only Ziehl-Neelsen staining, not from fluorescent SSM methods.

#### Mycobacterial Culture

The clinical samples can be used for mycobacterial culture (10^2^ bacilli•mL^-1^ of sputum); cultivation is still the gold standard for TB diagnosis. *M. tuberculosis* is usually cultured on a solid medium, where it can be further identified and tested for drug sensitivity, providing to clinicians an effective antibacterial treatment guidance (Kenaope et al., 2020). The liquid culture systems such as BACTEC MGIT 960, VersaTREK, and MB/BacT Alert 3D allow the detection of *M. tuberculosis* in a few days. The BACTEC MGIT 960 automated culture system monitors the oxygen quenching fluorescence, and the signal is detected once the mycobacteria grow in the tube. Hasan et al. found that MGIT 960 is effective in the quick detection of mycobacteria and early TB diagnosis than L-J solid medium (Hasan et al., 2013). The VersaTREK system is sensitive to pressure variation; thus, it detects the growth of the inoculated specimen by measuring the pressure change above the broth medium (Espasa et al., 2012). The MB / BacT Alert 3D system uses a colorimetric carbon dioxide sensor to detect the growth of *M. tuberculosis* (Piersimoni et al., 2001). Considering the slow growth of the *M. tuberculosis* complex (MTBC), most cultures positive for MTCB occur at least in 1 week, while a culture negative for MTCB occurs in 8 weeks (Lee et al., 2003).

### Molecular Biology Diagnostic Techniques

#### Xpert MTB/RIF

GeneXpert MTB/RIF is the most widely used detection method in molecular diagnostics. It is a semi-nested real-time fluorescent PCR for the detection of *M. tuberculosis* and rifampin resistance simultaneously. The Xpert MTB/RIF Ultra developed based on Xpert MTB/RIF increases two different multi-copy amplification targets and a larger DNA reaction chamber (World Health Organization, 2017). The limit for Xpert Ultra is increased to 15.6 CFU/ml compared to the detection limit of 112.6 CFU/ml of Xpert MTB/RIF (Chakravorty et al., 2017). This technology directly detects MTBC DNA in sputum or concentrated sputum deposits as well as rifampin resistance, producing results within 2 hours (Bodmer and Ströhle, 2012). In December 2010, the World Health Organization recommended Xpert MTB/RIF in the diagnosis of TB and drug resistance, especially in HIV patients and suspected patients with multidrug-resistant TB (WHO Guidelines Approved by the Guidelines Review Committee, 2011). In consideration of high demands for professional testing personnel and supporting infrastructure, the primary medical institutions have difficulties to meet the above requirements for Xpert MTB/RIF and ensure the quality of test results (Gidado et al., 2019).

#### Loop-Mediated Isothermal Amplification

Loop-mediated isothermal amplification is a type of Nucleic Acid Amplification Test that employs DNA polymerase and a set of specially designed primers to detect the presence of pathogenic DNA from a patient sample. The SS-LAMP is specially designed with a set of six specific primers to identify eight different regions on the MTBC-specific repeat insertion sequence 6,110 (IS6110), which is qualified to directly detect the DNA of MTBC from liquefied sputum samples (Bentaleb et al., 2016). A validation study of the method was performed using 157 liquefied sputum specimens from Moroccan suspected TB patients. SS-LAMP analysis is faster, with a specificity of 99.14% and a sensitivity of 82.93% compared with the conventional L-J solid culture method. LAMP method is suitable for areas where medical resources are relatively scarce.

#### Digital PCR

Digital PCR (dPCR) is a new type of nucleic acid quantification technology that requires very small amounts of target molecules, and it performs the absolute quantification without the need for a standard curve (Kuypers and Jerome, 2017). Therefore, dPCR is precise and sensitive, and most importantly, it detects single copies of DNA (Nyaruaba et al., 2019). The dPCR samples can be sputum, blood, formalin fixed paraffin embedded tissue, and exhaled breath. The drug sensitivity testing can also be performed by this method (Surat et al., 2014; Ushio et al., 2016; Patterson et al., 2017; Yang et al., 2017a; Luo et al., 2019; Cao et al., 2020; Cho et al., 2020). IS6110 is a common target for dPCR amplification, but when combined with IS1081 and IS6110, the dPCR sensitivity is higher than IS6110 qPCR, thus improving the diagnosis of smear-negative TB (Lyu et al., 2020). This method has been demonstrated as useful for studying in the case of lung, extrapulmonary, latent TB infection, and active TB, though more prone to error in the hands of inexperienced users (Nyaruaba et al., 2019).

### Immunological Diagnostic Techniques

#### Tuberculin Skin Test and Interferon-γ Release Assay

The tuberculin skin test and the subsequent widely used tuberculin protein derivative test play an important role in the auxiliary diagnosis of TB, especially in pediatrics TB. However, these approaches are neither able to effectively distinguish the positive results due to BCG vaccination or *M. tuberculosis* infection, nor provide reliable results for potential immunocompromised TB patients. The most widely studied antigens in *M. tuberculosis* include CFP-10, ESAT-6, Ag85A, Ag85B, CFP-7, and PPE18 (Fan et al., 2017; Ren et al., 2018). The interferon-γ release assay helps in the diagnosis of TB by detecting the secretion of IFN-γ by sampled lymphocytes after stimulation with ESAT-6 and CFP-10 antigens, which are quite specific for *M. tuberculosis*. Although the analytical performance of the two commercial kits is different, they have quite similar sensitivity in diagnosing latent and active TB infection. The immunological methods have certain limitations when dealing with HIV patients, immunocompromised adults, as well as children (Ayubi et al., 2016; Benachinmardi et al., 2019), and it requires professional knowledge and certain equipment.

#### Immuno-PCR

The Immuno-PCR (I-PCR) assay detects potential mycobacterial antigens and circulating antibodies in the body fluids of TB patients, thus, it can be used as a new diagnostic approach for TB. I-PCR based on magnetic beads (MBs)/ gold nanoparticles (GNPs) in liquid form produces a reduced background signal, and the automated one-step I-PCR also shortens the detection time (Mehta et al., 2017). Singh et al. designed an I-PCR (MB-GNP-I-PCR) detection kit based on MBs coupled with GNP to detect the early secreted antigen ESAT-6 (Singh et al., 2018). Dahiya et al. designed a GNP-RT-I-PCR analysis based on GNPs to detect the CFP-10 protein of *M. tuberculosis* in clinical samples of TB patients (Dahiya et al., 2020a). The sensitivity was 83.7 and 76.2%, and the specificity was 93.5 and 93.8% in 49 cases of pulmonary TB and 42 cases of extrapulmonary TB, respectively (*n* = 63). The MB coupled AuNP-based I-PCR (MB-AuNP-I-PCR) method has been designed to detect *M. tuberculosis* MPT64 and CFP-10 proteins in the body fluids of TB patients, and the results showed that the sensitivity of MB-AuNP-I-PCR in smear-negative pulmonary TB and extrapulmonary TB patients was significantly higher than that of Magneto-ELISA and GeneXpert analysis (Dahiya et al., 2020b).

The PCR-ELISA developed by Zhou et al. detects mutations in the rpoB, katG, and inhA genes caused by rifampicin and isoniazid resistance and predicts the drug susceptibility of clinical isolates of *M. tuberculosis* (Zhou et al., 2020). Ultrasensitive ELISA combines ELISA with a thionicotinamide-adenine dinucleotide cycling method, which rapidly achieves high sensitivity for diagnosing TB without *M. tuberculosis* culture (Watabe et al., 2014). MPT64 protein is specifically secreted only from live *M. tuberculosis* when the bacteria are heated (46°C); thus, ultrasensitive ELISA only detects the MPT64 protein secreted by live *M. tuberculosis* (Wang et al., 2020; Cao et al., 2021), and the sensitivity is almost the same as that of smear microscopy and Xpert MTB/RIF.

#### Lateral Flow Urine Lipoarabinomannan Assay

Lipoarabinomannan is a key lipopolysaccharide and pathogenic factor present in the cell wall of mycobacteria, with a representative structural epitope of *M. tuberculosis* (Sarkar et al., 2014). LF-LAM has high sensitivity in the diagnosis of TB in patients co-infected with HIV, especially those with low CD4 counts. WHO recommends the use of LF-LAM to diagnose and screen active TB in HIV-infected patients (World Health Organization, 2015). However, the sensitivity and specificity of LAM-based tests still need further population-scale studies to confirm the diagnostic potential (Sigal et al., 2018).

## New Promising Diagnostic Techniques

### Chemical Probe Methods

The chemical probes using radiology or optical imaging possess the ability of real-time quantification of bacteria and other infectious agents, providing an alternative method to culture (Heuts et al., 2009; Andreu et al., 2012). Multiple options are available for chemical probes, including fluorescence, chemiluminescence, light scattering, and radioactivity. Fluorescence is the most popular approach, since it is easily captured by fluorescence scanners/plate readers, time-lapse fluorescence microscopes, ultra-high-resolution microscopes, and flow cytometers (Hira et al., 2020).

#### Cell Envelope-Dependent Probes

Mycobacteria possess a distinct extracellular structure. The arabinogalactan, long-chain mycolic acid, and trehalose-enriched glycolipid contribute to form the unique membrane layer. The trehalose mycosyl esterase located on the cell membrane possesses conservative substrate specificity, which allows the exogenous addition of synthetic probes (FITC-trehalose) for a specific incorporation into bacteria. This specific incorporation is the key in the detection of bacterial growth through fluorescent labeling. The FITC-trehalose sensitively detects *M. tuberculosis* in a macrophage infection model (Backus et al., 2011). The 4-N,N-dimethylamino-1,8-naphthalimide conjugated trehalose probe (DMN-Tre) sensitively detects *M. tuberculosis* in sputum samples of TB patients; the fluorescence intensity is significantly enhanced (fluorescence intensity increase >700 fold) when the transition from aqueous to hydrophobic environment occurs. DMN-Tre labeling enabled the rapid, no-wash visualization of mycobacterial and corynebacterial species (<1 h) (Kamariza et al., 2018). DMN-Tre specifically detects metabolically active mycobacteria and corynebacteria, which is useful for investigating the drug response after treatment.

DLF-1 is a high affinity stoichiometric probe for the D-Ala-D-Ala motif of bacterial peptidoglycan. The fluorescent probe directly labels the cell wall components of *M. tuberculosis*, representing an alternative approach for a rapid quantitative analysis of active and dormant *M. tuberculosis in vitro* and *in vivo* (Yang et al., 2016). The D-Ala-D-Ala motif is also present in other bacterial strains besides *M. tuberculosis*; thus, DLF-1 cannot be specifically used to label *M. tuberculosis*.

#### BlaC-Specific Fluorogenic Probes

BlaC is an Ambler Class A β-lactamase, highly conserved among clinical isolates of *M. tuberculosis*, which effectively hydrolyzes β-lactam antibiotics (Kwon et al., 1995). The crystal structure of BlaC indicates the presence of an unusual glycines within the BlaC active site, which makes *M. tuberculosis* β-lactamase unique (Wang et al., 2006), thus becoming a biomarker for *M. tuberculosis* detection.

Xie et al. developed a fluorescent probe for *M. tuberculosis* using the flexible substrate-specific loop of BlaC enzyme and introducing a methoxyl substituent at the position 7 of the lactam ring, which made it 1,000 times more selective for BlaC than TEM-1 β-lactamase (Xie et al., 2012). The fluorescence intensity is enhanced 100–200 fold when BlaC is activated, which is useful for reducing false positives. The green fluorescent probe CDG-OME was developed for the sensitivity and specificity of detecting an extremely small number of live pathogens in the sputum of patients within 10 min, even in unprocessed sputum, making it a rapid, low-cost diagnostic tool for TB. On the basis of this work, Cheng et al. designed a CDG-3 probe with the substitution of the cyclopropane ring at the position 2 in addition to the substitution of the methoxide ring at the position 7, and its selectivity to BlaC is 120,000 times greater than TEM-1 β-lactamase (Cheng et al., 2014). CDG-3 detected *M. tuberculosis* within 1 h in a trial with 50 clinical samples, with a sensitivity and specificity of 90 and 73%, respectively. It is worth noting that signal diffusion problems compromised single *M. tuberculosis* labeling if the fluorescent probes only recognized BlaC; the ability of BlaC labeling to detect cell viability or drug sensitivity has not yet been reported. Cheng et al. further developed a dual-target fluorescent probe CDG-DNB targeting BlaC and DprE1; BlaC hydrolyzes the lactam ring to activate the fluorophore, while DprE1 covalently bind to an anchor element for fluorescence fixation (Cheng et al., 2018). The combination of BlaC and DprE1 facilitates *M. tuberculosis* fluorescent labeling. The dual targeting probe CDG-DNB specifically and accurately labels the single live *M. tuberculosis* in less than 1 h, and the specificity of CDG-DNB probe has been demonstrated, since it only selectively labels *M. tuberculosis* among other bacterial species (including 43 NTM).

Sule et al. synthesized CDG-3, which is a fluorescent reporter enzyme substrate specific for BlaC (REF) and developed a TB diagnostic method used in sputum specimens (Sule et al., 2016). This method detects *M. tuberculosis* also in clinical samples that are contaminated by other bacteria. The CDG-3 probe has high selectivity to BlaC, and is used to measure the levels of enzymes and *M. tuberculosis* in sputum. The sensitivity and specificity for CDG-3 according to the ROC curve were 88.1 and 86.1% in 160 clinical specimens from potential TB patients, with a negative predictive value of 93%; thus, it could be used to predict potential TB patients (Sule et al., 2019). BlaC is secreted by the Tat secretion system in live *M. tuberculosis* (McDonough et al., 2005); thus, the BlaC-specific reporter enzyme fluorescence test is suitable for the evaluation of the treatment results and phenotypic drug susceptibility. Yang et al. synthesized a novel REF substrate CNIR800 with the near-infrared (NIR) fluorescent dye IRDye 800CW (Yang et al., 2017b). The quenching agent of IRDye 800CW is bond to a lactam ring, which is hydrolyzed by BlaC. The emission wavelength of CNIR800 is 795 nm, which significantly improves the signal to noise ratio for *M. tuberculosis* detection. The detection threshold of CNIR800 is ~100 CFU *in vitro* and < 1,000 CFU in the lungs of mice. The fluorescence signal of CNIR800 produced by cleavage reaches its maximum level 4–6 h after its administration in live animals, allowing the accurate assessment of the efficacy of antituberculosis drugs.

#### Probes Dependent on Sulfatase, Esterase, Protease, and Nitroreductase

Beatty et al. developed a sulfate activation probe (7-hydroxy-9H -(1, 3-dichloro-9, 9-dimethylacridin-2-ketone)-sulfate targeting mycobacterial sulfotransferases (Beatty et al., 2013) and conserved sulfatases (Mougous et al., 2002). Mycobacteria have unique sulfatase fingerprints, which can be used to determine mycobacterial species and lineage, and this probe has the potential to detect TB. Recently, the highly conserved esterase activity of the MTBC has been discovered (Tallman and Beatty, 2015). Tallman et al. synthesized new C4- and C8-masked probes using four-carbon (C4) and eight-carbon (C8) acylloxymethyl ether derivatives of long-chain fluorescent substrates to analyze the lysates of macrophages infected with *M. tuberculosis*, and identified the patterns of *M. tuberculosis* esterase and lipase bands (Tallman et al., 2016).

Babin et al. developed a rapid and economical chemiluminescent protease Hip1 probe (FLASH) (Babin et al., 2021). The FLASH probe is cleaved by *M. tuberculosis* protease Hip1; then, the aniline linker undergoes spontaneous elimination, releasing the activated phenoxy-dioxetane luminophore. FLASH allows the quantification of active Hip1, thus detecting and quantifying *M. tuberculosis* in 1 h, useful in the analysis of clinical sputum samples. FLASH also distinguish live from dead cells, allowing the monitoring the drug susceptibility of clinical *M. tuberculosis* isolates. Mu et al. developed a nitrooxidoreductase Rv2466c-dependent fluorescent probe (Mu et al., 2019). The small molecule mycothiol (MSH) of *M. tuberculosis* binds to Rv2466c; its sulfhydryl group forms a disulfide bond with the Cys19 to activate Rv2466c, allowing the entrance of the coumarin-based nitrofuranyl calanolides (NFCs) into Rv2466c and interact with W21, N51, and Y61 of Rv2466c to form the Rv2466c-mycothiol-NFC ternary complex. Rv2466c reduces the nitro group of NFCs to an amino group, generating high level of fluorescence, thus potentially useful for a rapid diagnosis and drug sensitivity test of clinical sputum samples.

### Clustered Regularly Interspaced Short Palindromic Repeats

CRISPR-Cas acts in a sequence-specific manner by recognizing and cleaving DNA or RNA, allowing an improved identification and validation of the target. The sensitivity and specificity of CRISPR-Cas are comparable to conventional PCR, but it does not require complicated equipment as PCR and is very cost-effective. The CRISPR-MTB detection system uses Cas12a endonuclease that recognizes double-stranded DNA and cuts it into single-stranded DNA and combines isothermal amplification technology to achieve nearly single copy level sensitivity, with the additional benefit of only requiring 500 μl of sample (Ai et al., 2019). However, these amplifications based on molecular biology diagnostic methods have high false positive rates and are unable to distinguish dead from live bacteria.

### Mass Spectrometry

Matrix-assisted laser desorption ionization-time of flight mass spectrometry (MALDI-TOF) is used to identify tuberculous complex and most atypical mycobacteria from cultures (Lotz et al., 2010; El Khéchine et al., 2011). The identification of *M. tuberculosis* from a L-J solid culture is superior to that from liquid culture, probably due to the interference exerted by certain components of the liquid medium. The combination of exhaled breath collected by a bioaerosol sampling system and high-resolution mass spectrometry is used to identify *M. tuberculosis* (Chen et al., 2020). Currently, the FDA-approved two MALDI-TOF platforms (namely MALDI Biotyper and Vitek MS, BioMérieux) are used to identify mycobacteria and a few other bacteria. The mass spectrometry approach can be performed in a routine clinical laboratory by the operator within a few minutes (Seng et al., 2010), although it requires expensive laboratory infrastructure and specialized staff, as well as shorts for an available extensive library for data alignment.

### Immunosensors

Immunosensors are often used to detect and quantify disease-related substances in clinical diagnosis due to their increased affinity to the antigen and antibody complex with great selectivity. Electrochemical immunosensors used in TB diagnosis are based on the use of monoclonal antibodies to detect specific proteins secreted by *M. tuberculosis* (Montoya et al., 2017; Mohd Bakhori et al., 2019; Peláez et al., 2020). they quantitatively monitor the electrical signals generated by the binding between antibodies and target molecules or antigens of *M. tuberculosis*. Moreover, the current nanomaterial immunosensor relies on the specific chemical, physical and electronic properties of the nanomaterials themselves to improve the performance of the sensing device and diagnose TB in real-time (Mohd Bakhori et al., 2019; Hatami et al., 2020; Kahng et al., 2020). Although biosensors are promising, portable, easy to operate, with no amplification steps, immunosensors did not achieve the same success as immunoassays(Shin et al., 2015). This is probably due to the detection limit of 10^3^ copies to diagnose *M. tuberculosis* (Jaroenram et al., 2020). In addition, problems occur to identify latent infections, as well as pediatric and immunocompromised HIV patients(MacGregor-Fairlie et al., 2020). Therefore, further efforts are needed to develop immunosensors that are effective in diagnosing TB.

### Next-Generation Sequencing Technology

Next-generation massively parallel sequencing allows to sequence millions of fragments simultaneously in each run and has been recently proposed to provide profiles of drug resistance within a single analysis of drug-resistant TB (Tafess et al., 2020). Drug susceptibility testing is achieved through targeted or whole genome sequencing methods (Papaventsis et al., 2017). The traditional whole genome sequencing of *M. tuberculosis* depends on bacterial culture (Iketleng et al., 2018), but the direct whole genome sequencing of the sputum developed by Doyle et al. bypasses the process of bacterial culture (Doyle et al., 2018), thus saving detection time, and significantly improving the speed of detecting drug resistance compared with the whole genome sequencing of MGIT or culture-based drug resistance phenotype test in clinical practice. However, the effective and convenient manufacturing of more DNA isolation devices, the cost of throughput sequencing, good databases, the high requirements of the laboratory infrastructure, and the need for specialized staff remain the main obstacles to the clinical application of next-generation sequencing technology (Smith et al., 2020).

## Conclusion

TB represented and still represents a continuous challenge to the global public health. In the last years, significant progress has been made in the development of TB diagnostics platforms However, TB is still diagnosed late or misdiagnosed, as well as properly monitored and treated, since the emergence of multidrug-resistant strains has further worsened the situation. In recent years, traditional methods for detecting mycobacteria have been continuously improved, and considerable efforts have been made for the development of new methods. Nevertheless, several key factors need to be further addressed to obtain techniques allowing a rapid TB diagnosis: (1) Diagnosis of extrapulmonary TB, TB in children, people with TB and HIV, and TB in pregnant women. (2) Tests for drug response or drug resistant TB. (3) Effective and affordable test materials. (4) Sensitive and specific methods of becoming positive for *M. tuberculosis* other than BCG vaccination. Therefore, more accurate, rapid, sensitive, selective, cost-effective diagnostic techniques and tools are needed for the recognition of positive cases and the detection of drug-resistant TB. The development, evaluation, and improvement of new diagnostic methods would be especially successful in clinical application. Thus, all the above continues to be a necessary area of research.

## Author Contributions

BD and ZH wrote the original draft. JZ reviewed and edited the manuscript with help from YL. XX contributed the schematic diagram. CW and JZ provided the conceptualization and fundings. All authors contributed to the article and approved the submitted version.

## Funding

This work was supported by the Technological Innovation and Development Project of Chengdu Bureau of Science and Technology (2021-YF05-01819-SN) and the Fundamental Research Funds for the Central Universities (YJ201985).

## Conflict of Interest

The authors declare that the research was conducted in the absence of any commercial or financial relationships that could be construed as a potential conflict of interest.

## Publisher’s Note

All claims expressed in this article are solely those of the authors and do not necessarily represent those of their affiliated organizations, or those of the publisher, the editors and the reviewers. Any product that may be evaluated in this article, or claim that may be made by its manufacturer, is not guaranteed or endorsed by the publisher.

## References

[ref1] Ai J. W. Zhou X. Xu T. Yang M. Chen Y. He G. Q.. (2019). CRISPR-based rapid and ultra-sensitive diagnostic test for mycobacterium tuberculosis. Emerg. Microbes Infect. 8, 1361–1369. doi: 10.1080/22221751.2019.1664939, PMID: 31522608 PMC6758691

[ref2] Andreu N. Fletcher T. Krishnan N. Wiles S. Robertson B. D. (2012). Rapid measurement of antituberculosis drug activity in vitro and in macrophages using bioluminescence. J. Antimicrob. Chemother. 67, 404–414. doi: 10.1093/jac/dkr472, PMID: 22101217 PMC3254196

[ref3] Ayubi E. Doosti-Irani A. Sanjari Moghaddam A. Sani M. Nazarzadeh M. Mostafavi E. (2016). The clinical usefulness of tuberculin skin test versus interferon-gamma release assays for diagnosis of latent tuberculosis in HIV patients: a meta-analysis. PLoS One 11:e0161983. doi: 10.1371/journal.pone.0161983, PMID: 27622293 PMC5021339

[ref4] Babin B. M. Fernandez-Cuervo G. Sheng J. Green O. Ordonez A. A. Turner M. L.. (2021). Chemiluminescent protease probe for rapid, sensitive, and inexpensive detection of live mycobacterium tuberculosis. ACS Cent Sci. 7, 803–814. doi: 10.1021/acscentsci.0c01345, PMID: 34079897 PMC8161474

[ref5] Backus K. M. Boshoff H. I. Barry C. S. Boutureira O. Patel M. K. D'Hooge F.. (2011). Uptake of unnatural trehalose analogs as a reporter for mycobacterium tuberculosis. Nat. Chem. Biol. 7, 228–235. doi: 10.1038/nchembio.539, PMID: 21378984 PMC3157484

[ref6] Beatty K. E. Williams M. Carlson B. L. Swarts B. M. Warren R. M. van Helden P. D.. (2013). Sulfatase-activated fluorophores for rapid discrimination of mycobacterial species and strains. Proc. Natl. Acad. Sci. U. S. A. 110, 12911–12916. doi: 10.1073/pnas.1222041110, PMID: 23878250 PMC3740907

[ref7] Benachinmardi K. K. Sangeetha S. Rao M. Prema R. (2019). Validation and clinical application of interferon-gamma release assay for diagnosis of latent tuberculosis infection in children. Int. J. Appl. Basic Med. Res. 9, 241–245. doi: 10.4103/ijabmr.IJABMR_86_19, PMID: 31681551 PMC6822318

[ref8] Ben-Selma W. Ben-Kahla I. Marzouk M. Ferjeni A. Ghezal S. Ben-Said M.. (2009). Rapid detection of mycobacterium tuberculosis in sputum by Patho-TB kit in comparison with direct microscopy and culture. Diagn. Microbiol. Infect. Dis. 65, 232–235. doi: 10.1016/j.diagmicrobio.2009.07.021, PMID: 19729264

[ref9] Bentaleb E. M. Abid M. El Messaoudi M. D. Lakssir B. Ressami E. M. Amzazi S.. (2016). Development and evaluation of an in-house single step loop-mediated isothermal amplification (SS-LAMP) assay for the detection of mycobacterium tuberculosis complex in sputum samples from Moroccan patients. BMC Infect. Dis. 16:517. doi: 10.1186/s12879-016-1864-9, PMID: 27677540 PMC5039794

[ref10] Bodmer T. Ströhle A. (2012). Diagnosing pulmonary tuberculosis with the Xpert MTB/RIF test. J. Vis. Exp. 62:e3547. doi: 10.3791/3547 PMC3598386 22508485

[ref11] Cao X. J. Li Y. P. Wang J. Y. Zhou J. Guo X. G. (2021). MPT64 assays for the rapid detection of mycobacterium tuberculosis. BMC Infect. Dis. 21:336. doi: 10.1186/s12879-021-06022-w, PMID: 33838648 PMC8035777

[ref12] Cao Z. Wu W. Wei H. Gao C. Zhang L. Wu C.. (2020). Using droplet digital PCR in the detection of mycobacterium tuberculosis DNA in FFPE samples. Int. J. Infect. Dis. 99, 77–83. doi: 10.1016/j.ijid.2020.07.045, PMID: 32738487

[ref13] Chakravorty S. Simmons A. M. Rowneki M. Parmar H. Cao Y. Ryan J.. (2017). The new Xpert MTB/RIF ultra: improving detection of mycobacterium tuberculosis and resistance to rifampin in an assay suitable for point-of-care testing. MBio 8, e00812–e00817. doi: 10.1128/mBio.00812-17 28851844 PMC5574709

[ref14] Chen D. Bryden W. A. Wood R. (2020). Detection of tuberculosis by the analysis of exhaled breath particles with high-resolution mass spectrometry. Sci. Rep. 10:7647. doi: 10.1038/s41598-020-64637-6, PMID: 32376992 PMC7203136

[ref15] Cheng Y. Xie J. Lee K. H. Gaur R. L. Song A. Dai T.. (2018). Rapid and specific labeling of single live mycobacterium tuberculosis with a dual-targeting fluorogenic probe. Sci. Transl. Med. 10:eaar4470. doi: 10.1126/scitranslmed.aar4470 30111644 PMC6314683

[ref16] Cheng Y. Xie H. Sule P. Hassounah H. Graviss E. A. Kong Y.. (2014). Fluorogenic probes with substitutions at the 2 and 7 positions of cephalosporin are highly BlaC-specific for rapid mycobacterium tuberculosis detection. Angew. Chem. Int. Ed. Engl. 53, 9360–9364. doi: 10.1002/anie.201405243, PMID: 24989449 PMC4499257

[ref17] Cho S. M. Shin S. Kim Y. Song W. Hong S. G. Jeong S. H.. (2020). A novel approach for tuberculosis diagnosis using exosomal DNA and droplet digital PCR. Clin. Microbiol. Infect. 26, 942.e1–942.e5. doi: 10.1016/j.cmi.2019.11.012 31760116

[ref18] Dahiya B. Prasad T. Singh V. Khan A. Kamra E. Mor P.. (2020a). Diagnosis of tuberculosis by nanoparticle-based immuno-PCR assay based on mycobacterial MPT64 and CFP-10 detection. Nanomedicine (Lond.) 15, 2609–2624. doi: 10.2217/nnm-2020-0258, PMID: 33090059

[ref19] Dahiya B. Sharma S. Khan A. Kamra E. Mor P. Sheoran A.. (2020b). Detection of mycobacterial CFP-10 (Rv3874) protein in tuberculosis patients by gold nanoparticle-based real-time immuno-PCR. Future Microbiol. 15, 601–612. doi: 10.2217/fmb-2019-0347, PMID: 32490745

[ref20] Doyle R. M. Burgess C. Williams R. Gorton R. Booth H. Brown J.. (2018). Direct whole-genome sequencing of sputum accurately identifies drug-resistant mycobacterium tuberculosis faster than MGIT culture sequencing. J. Clin. Microbiol. 56, e00666–e00618. doi: 10.1128/JCM.00666-18 29848567 PMC6062781

[ref21] El Khéchine A. Couderc C. Flaudrops C. Raoult D. Drancourt M. (2011). Matrix-assisted laser desorption/ionization time-of-flight mass spectrometry identification of mycobacteria in routine clinical practice. PLoS One 6:e24720. doi: 10.1371/journal.pone.0024720, PMID: 21935444 PMC3172293

[ref22] Erokhina M. V. Lepekha L. N. Voronezhskaya E. E. Nezlin L. P. Avdienko V. G. Ergeshov A. E. (2019). Application of laser scanning confocal microscopy for the visualization of *M. tuberculosis* in lung tissue samples with weak Ziehl-Neelsen staining. J. Clin. Med. 8:1185. doi: 10.3390/jcm8081185 PMC6723956 31394889

[ref23] Espasa M. Salvadó M. Vicente E. Tudó G. Alcaide F. Coll P.. (2012). Evaluation of the VersaTREK system compared to the Bactec MGIT 960 system for first-line drug susceptibility testing of mycobacterium tuberculosis. J. Clin. Microbiol. 50, 488–491. doi: 10.1128/JCM.06432-11, PMID: 22135258 PMC3264154

[ref24] Fan J. Zhang H. Nguyen D. T. Lyon C. J. Mitchell C. D. Zhao Z.. (2017). Rapid diagnosis of new and relapse tuberculosis by quantification of a circulating antigen in HIV-infected adults in the greater Houston metropolitan area. BMC Med. 15:188. doi: 10.1186/s12916-017-0952-z, PMID: 29089034 PMC5664577

[ref25] Gidado M. Nwokoye N. Ogbudebe C. Nsa B. Nwadike P. Ajiboye P.. (2019). Assessment of GeneXpert MTB/RIF performance by type and level of health-care facilities in Nigeria. Niger. Med. J. 60, 33–39. doi: 10.4103/nmj.NMJ_12_19, PMID: 31413433 PMC6677003

[ref26] Hasan M. Munshi S. K. Banu Momi M. S. Rahman F. Noor R. (2013). Evaluation of the effectiveness of BACTEC MGIT 960 for the detection of mycobacteria in Bangladesh. Int. J. Mycobacteriol. 2, 214–219. doi: 10.1016/j.ijmyco.2013.09.001, PMID: 26786125

[ref27] Hatami Z. Ragheb E. Jalali F. Tabrizi M. A. Shamsipur M. (2020). Zinc oxide-gold nanocomposite as a proper platform for label-free DNA biosensor. Bioelectrochemistry 133:107458. doi: 10.1016/j.bioelechem.2020.107458, PMID: 32006859

[ref28] Heuts F. Carow B. Wigzell H. Rottenberg M. E. (2009). Use of non-invasive bioluminescent imaging to assess mycobacterial dissemination in mice, treatment with bactericidal drugs and protective immunity. Microbes Infect. 11, 1114–1121. doi: 10.1016/j.micinf.2009.08.005, PMID: 19682599

[ref29] Hira J. Uddin M. J. Haugland M. M. Lentz C. S. (2020). From differential stains to next generation physiology: chemical probes to visualize bacterial cell structure and physiology. Molecules 25:4949. doi: 10.3390/molecules25214949, PMID: 33114655 PMC7663024

[ref30] Iketleng T. Lessells R. Dlamini M. T. Mogashoa T. Mupfumi L. Moyo S.. (2018). Mycobacterium tuberculosis next-generation whole genome sequencing: opportunities and challenges. Tuberc Res Treat. 2018, 1–8. doi: 10.1155/2018/1298542 PMC6304523 30631597

[ref31] Jaroenram W. Kampeera J. Arunrut N. Karuwan C. Sappat A. Khumwan P.. (2020). Graphene-based electrochemical genosensor incorporated loop-mediated isothermal amplification for rapid on-site detection of mycobacterium tuberculosis. J. Pharm. Biomed. Anal. 186:113333. doi: 10.1016/j.jpba.2020.113333, PMID: 32402994

[ref32] Kahng S. J. Soelberg S. D. Fondjo F. Kim J. H. Furlong C. E. Chung J. H. (2020). Carbon nanotube-based thin-film resistive sensor for point-of-care screening of tuberculosis. Biomed. Microdevices 22:50. doi: 10.1007/s10544-020-00506-3, PMID: 32725281

[ref33] Kamariza M. Shieh P. Ealand C. S. Peters J. S. Chu B. Rodriguez-Rivera F. P.. (2018). Rapid detection of mycobacterium tuberculosis in sputum with a solvatochromic trehalose probe. Sci. Transl. Med. 10:aam6310. doi: 10.1126/scitranslmed.aam6310 PMC5985656 29491187

[ref34] Kenaope L. Ferreira H. Seedat F. Otwombe K. Martinson N. A. Variava E. (2020). Sputum culture and drug sensitivity testing outcome among X-pert mycobacterium tuberculosis/rifampicin-positive, rifampicin-resistant sputum: a retrospective study – not all rifampicin resistance is multi-drug resistant. J. Glob. Antimicrob. Resist. 21, 434–438. doi: 10.1016/j.jgar.2019.11.008, PMID: 31733411

[ref35] Kuypers J. Jerome K. R. (2017). Applications of digital PCR for clinical microbiology. J. Clin. Microbiol. 55, 1621–1628. doi: 10.1128/JCM.00211-17, PMID: 28298452 PMC5442518

[ref36] Kwon H. H. Tomioka H. Saito H. (1995). Distribution and characterization of beta-lactamases of mycobacteria and related organisms. Tuber. Lung Dis. 76, 141–148. doi: 10.1016/0962-8479(95)90557-X, PMID: 7780097

[ref37] Lee J. J. Suo J. Lin C. B. Wang J. D. Lin T. Y. Tsai Y. C. (2003). Comparative evaluation of the BACTEC MGIT 960 system with solid medium for isolation of mycobacteria. Int. J. Tuberc. Lung Dis. 7, 569–574. doi: 10.1080/01902140390197095 12797700

[ref38] Lönnroth K. Castro K. G. Chakaya J. M. Chauhan L. S. Floyd K. Glaziou P.. (2010). Tuberculosis control and elimination 2010-50: cure, care, and social development. Lancet 375, 1814–1829. doi: 10.1016/S0140-6736(10)60483-7, PMID: 20488524

[ref39] Lotz A. Ferroni A. Beretti J. L. Dauphin B. Carbonnelle E. Guet-Revillet H.. (2010). Rapid identification of mycobacterial whole cells in solid and liquid culture media by matrix-assisted laser desorption ionization-time of flight mass spectrometry. J. Clin. Microbiol. 48, 4481–4486. doi: 10.1128/JCM.01397-10, PMID: 20943874 PMC3008439

[ref40] Luo J. Luo M. Li J. Yu J. Yang H. Yi X.. (2019). Rapid direct drug susceptibility testing of mycobacterium tuberculosis based on culture droplet digital polymerase chain reaction. Int. J. Tuberc. Lung Dis. 23, 219–225. doi: 10.5588/ijtld.18.0182, PMID: 30808455

[ref41] Lyu L. Li Z. Pan L. Jia H. Sun Q. Liu Q.. (2020). Evaluation of digital PCR assay in detection of M.tuberculosis IS6110 and IS1081 in tuberculosis patients plasma. BMC Infect. Dis. 20:657. doi: 10.1186/s12879-020-05375-y 32894079 PMC7487892

[ref42] MacGregor-Fairlie M. Wilkinson S. Besra G. S. Goldberg O. P. (2020). Tuberculosis diagnostics: overcoming ancient challenges with modern solutions. Emerg. Top. Life Sci. 4, 423–436. doi: 10.1042/ETLS20200335, PMID: 33258943 PMC7733669

[ref43] McDonough J. A. Hacker K. E. Flores A. R. Pavelka M. S. Jr. Braunstein M. (2005). The twin-arginine translocation pathway of mycobacterium smegmatis is functional and required for the export of mycobacterial beta-lactamases. J. Bacteriol. 187, 7667–7679. doi: 10.1128/JB.187.22.7667-7679.2005, PMID: 16267291 PMC1280313

[ref44] Mehta P. K. Dahiya B. Sharma S. Singh N. Dharra R. Thakur Z.. (2017). Immuno-PCR, a new technique for the serodiagnosis of tuberculosis. J. Microbiol. Methods 139, 218–229. doi: 10.1016/j.mimet.2017.05.009, PMID: 28527886

[ref45] Mohd Bakhori N. Yusof N. A. Abdullah J. Wasoh H. Ab Rahman S. K. Abd Rahman S. F. (2019). Surface enhanced CdSe/ZnS QD/SiNP electrochemical Immunosensor for the detection of mycobacterium tuberculosis by combination of CFP10-ESAT6 for better diagnostic specificity. Materials 13:149. doi: 10.3390/ma13010149, PMID: 31906075 PMC6982155

[ref46] Montoya A. March C. Montagut Y. J. Moreno M. J. Manclus J. J. Arnau A.. (2017). A high fundamental frequency (HFF)-based QCM Immunosensor for tuberculosis detection. Curr. Top. Med. Chem. 17, 1623–1630. doi: 10.2174/1568026617666161104105210, PMID: 27823567

[ref47] Mougous J. D. Green R. E. Williams S. J. Brenner S. E. Bertozzi C. R. (2002). Sulfotransferases and sulfatases in mycobacteria. Chem. Biol. 9, 767–776. doi: 10.1016/S1074-5521(02)00175-8, PMID: 12144918

[ref48] Mu R. Kong C. Yu W. Wang H. Ma Y. Li X.. (2019). Nitrooxidoreductase Rv2466c-dependent fluorescent probe for mycobacterium tuberculosis diagnosis and drug susceptibility testing. ACS Infect Dis. 5, 949–961. doi: 10.1021/acsinfecdis.9b00006, PMID: 30916931

[ref49] Nyaruaba R. Mwaliko C. Kering K. K. Wei H. (2019). Droplet digital PCR applications in the tuberculosis world. Tuberculosis (Edinb.) 117, 85–92. doi: 10.1016/j.tube.2019.07.001, PMID: 31378274

[ref50] Papaventsis D. Casali N. Kontsevaya I. Drobniewski F. Cirillo D. M. Nikolayevskyy V. (2017). Whole genome sequencing of mycobacterium tuberculosis for detection of drug resistance: a systematic review. Clin. Microbiol. Infect. 23, 61–68. doi: 10.1016/j.cmi.2016.09.008, PMID: 27665704

[ref51] Patterson B. Morrow C. Singh V. Moosa A. Gqada M. Woodward J.. (2017). Detection of mycobacterium tuberculosis bacilli in bio-aerosols from untreated TB patients. Gates Open Res. 1:11. doi: 10.12688/gatesopenres.12758.1 29355225 PMC5757796

[ref52] Peláez E. C. Estevez M. C. Mongui A. Menéndez M. C. Toro C. Herrera-Sandoval O. L.. (2020). Detection and quantification of HspX antigen in sputum samples using plasmonic biosensing: toward a real point-of-care (POC) for tuberculosis diagnosis. ACS Infect. Dis. 6, 1110–1120. doi: 10.1021/acsinfecdis.9b00502, PMID: 32233503

[ref53] Piersimoni C. Scarparo C. Callegaro A. Tosi C. P. Nista D. Bornigia S.. (2001). Comparison of MB/Bact alert 3D system with radiometric BACTEC system and Löwenstein-Jensen medium for recovery and identification of mycobacteria from clinical specimens: a multicenter study. J. Clin. Microbiol. 39, 651–657. doi: 10.1128/JCM.39.2.651-657.2001, PMID: 11158124 PMC87793

[ref54] Ren N. JinLi J. Chen Y. Zhou X. Wang J. Ge P.. (2018). Identification of new diagnostic biomarkers for mycobacterium tuberculosis and the potential application in the serodiagnosis of human tuberculosis. Microb. Biotechnol. 11, 893–904. doi: 10.1111/1751-7915.13291, PMID: 29952084 PMC6116745

[ref55] Sarkar P. Biswas D. Sindhwani G. Rawat J. Kotwal A. Kakati B. (2014). Application of lipoarabinomannan antigen in tuberculosis diagnostics: current evidence. Postgrad. Med. J. 90, 155–163. doi: 10.1136/postgradmedj-2013-132053, PMID: 24429376

[ref56] Seng P. Rolain J. M. Fournier P. E. La Scola B. Drancourt M. Raoult D.. (2010). MALDI-TOF-mass spectrometry applications in clinical microbiology. Future Microbiol. 5, 1733–1754. doi: 10.2217/fmb.10.127 21133692

[ref57] Shin Y. Perera A. P. Tang W. Y. Fu D. L. Liu Q. Sheng J. K.. (2015). A rapid amplification/detection assay for analysis of mycobacterium tuberculosis using an isothermal and silicon bio-photonic sensor complex. Biosens. Bioelectron. 68, 390–396. doi: 10.1016/j.bios.2015.01.030, PMID: 25615836

[ref58] Sigal G. B. Pinter A. Lowary T. L. Kawasaki M. Li A. Mathew A.. (2018). A novel sensitive immunoassay targeting the 5-Methylthio-d-xylofuranose-lipoarabinomannan epitope meets the WHO’s performance target for tuberculosis diagnosis. J. Clin. Microbiol. 56, e01338–e01318. doi: 10.1128/JCM.01338-18 30257899 PMC6258851

[ref59] Singh N. Dahiya B. Radhakrishnan V. S. Prasad T. Mehta P. K. (2018). Detection of mycobacterium tuberculosis purified ESAT-6 (Rv3875) by magnetic bead-coupled gold nanoparticle-based immuno-PCR assay. Int. J. Nanomedicine 13, 8523–8535. doi: 10.2147/IJN.S181052, PMID: 30587975 PMC6296691

[ref60] Smith C. Halse T. A. Shea J. Modestil H. Fowler R. C. Musser K. A.. (2020). Assessing nanopore sequencing for clinical diagnostics: a comparison of next-generation sequencing (NGS) methods for mycobacterium tuberculosis. J. Clin. Microbiol. 59, e00583–e00520. doi: 10.1128/JCM.00583-20 33055186 PMC7771457

[ref61] Sua L. F. Bolaños J. E. Maya J. Sánchez A. Medina G. Zúñiga-Restrepo V.. (2021). Detection of mycobacteria in paraffin-embedded Ziehl-Neelsen-stained tissues using digital pathology. Tuberculosis 126:102025. doi: 10.1016/j.tube.2020.102025, PMID: 33254011

[ref62] Sule P. Tilvawala R. Behinaein P. Walkup G. K. Cirillo J. D. (2016). New directions using reporter enzyme fluorescence (REF) as a tuberculosis diagnostic platform. Tuberculosis 101, S78–S82. doi: 10.1016/j.tube.2016.09.009 PMC5183490 27729258

[ref63] Sule P. Tilvawala R. Mustapha T. Hassounah H. Noormohamed A. Kundu S.. (2019). Rapid tuberculosis diagnosis using reporter enzyme fluorescence. J. Clin. Microbiol. 57, e01462–e01419. doi: 10.1128/JCM.01462-19 31511338 PMC6879286

[ref64] Surat G. Wallace W. A. Laurenson I. F. Seagar A. L. (2014). Rapid real-time PCR for detection of mycobacterium tuberculosis complex DNA in formalin-fixed paraffin embedded tissues: 16% of histological 'sarcoid' may contain such DNA. J. Clin. Pathol. 67, 1084–1087. doi: 10.1136/jclinpath-2014-202307, PMID: 25170093

[ref65] Tafess K. Ng T. T. L. Lao H. Y. Leung K. S. S. Tam K. K. G. Rajwani R.. (2020). Targeted-sequencing workflows for comprehensive drug resistance profiling of mycobacterium tuberculosis cultures using two commercial sequencing platforms: comparison of analytical and diagnostic performance, turnaround time, and cost. Clin. Chem. 66, 809–820. doi: 10.1093/clinchem/hvaa092, PMID: 32402055

[ref66] Tallman K. R. Beatty K. E. (2015). Far-red fluorogenic probes for esterase and lipase detection. Chembiochem 16, 70–75. doi: 10.1002/cbic.201402548, PMID: 25469918 PMC5096734

[ref67] Tallman K. R. Levine S. R. Beatty K. E. (2016). Profiling esterases in mycobacterium tuberculosis using far-red fluorogenic substrates. ACS Chem. Biol. 11, 1810–1815. doi: 10.1021/acschembio.6b00233, PMID: 27177211 PMC7880554

[ref68] Ushio R. Yamamoto M. Nakashima K. Watanabe H. Nagai K. Shibata Y.. (2016). Digital PCR assay detection of circulating mycobacterium tuberculosis DNA in pulmonary tuberculosis patient plasma. Tuberculosis (Edinb.) 99, 47–53. doi: 10.1016/j.tube.2016.04.004, PMID: 27450004

[ref69] Wang F. Cassidy C. Sacchettini J. C. (2006). Crystal structure and activity studies of the mycobacterium tuberculosis beta-lactamase reveal its critical role in resistance to beta-lactam antibiotics. Antimicrob. Agents Chemother. 50, 2762–2771. doi: 10.1128/AAC.00320-06, PMID: 16870770 PMC1538687

[ref70] Wang W. H. Takeuchi R. Jain S. H. Jiang Y. H. Watanuki S. Ohtaki Y.. (2020). A novel, rapid (within hours) culture-free diagnostic method for detecting live mycobacterium tuberculosis with high sensitivity. EBioMedicine 60:103007. doi: 10.1016/j.ebiom.2020.103007, PMID: 32949995 PMC7501073

[ref71] Watabe S. Kodama H. Kaneda M. Morikawa M. Nakaishi K. Yoshimura T.. (2014). Ultrasensitive enzyme-linked immunosorbent assay (ELISA) of proteins by combination with the thio-NAD cycling method. Biophysics 10, 49–54. doi: 10.2142/biophysics.10.49, PMID: 27493498 PMC4629663

[ref72] WHO Guidelines Approved by the Guidelines Review Committee (2011) Policy Statement: Automated Real-Time Nucleic Acid Amplification Technology for Rapid and Simultaneous Detection of Tuberculosis and Rifampicin Resistance: Xpert MTB/RIF System. Geneva: World Health Organization. 26158191

[ref73] World Health Organization (2015) The Use of Lateral Flow Urine Lipoarabinomannan Assay (LF-LAM) for the Diagnosis and Screening of Active Tuberculosis in People Living with HIV: Policy Guidance. Geneva: World Health Organization.

[ref74] World Health Organization (2017) WHO Meeting Report of a Technical Expert Consultation: Non- inferiority Analysis of Xpert MTB/RIF Ultra Compared to Xpert MTB/RIF. Geneva: World Health Organization.

[ref75] Xie H. Mire J. Kong Y. Chang M. Hassounah H. A. Thornton C. N.. (2012). Rapid point-of-care detection of the tuberculosis pathogen using a BlaC-specific fluorogenic probe. Nat. Chem. 4, 802–809. doi: 10.1038/nchem.1435, PMID: 23000993 PMC4136554

[ref76] Yang D. Ding F. Mitachi K. Kurosu M. Lee R. E. Kong Y. (2016). A fluorescent probe for detecting mycobacterium tuberculosis and identifying genes critical for cell entry. Front. Microbiol. 7:2021. doi: 10.3389/fmicb.2016.02021 28066347 PMC5168438

[ref77] Yang J. Han X. Liu A. Bai X. Xu C. Bao F.. (2017a). Use of digital droplet PCR to detect mycobacterium tuberculosis DNA in whole blood-derived DNA samples from patients with pulmonary and Extrapulmonary tuberculosis. Front. Cell. Infect. Microbiol. 7:369. doi: 10.3389/fcimb.2017.00369, PMID: 28848722 PMC5554497

[ref78] Yang H. J. Kong Y. Cheng Y. Janagama H. Hassounah H. Xie H.. (2017b). Real-time imaging of mycobacterium tuberculosis, using a novel near-infrared fluorescent substrate. J. Infect. Dis. 215, 405–414. doi: 10.1093/infdis/jiw298, PMID: 27421748 PMC6061879

[ref79] Zhou Y. C. He S. M. Wen Z. L. Zhao J. W. Song Y. Z. Zhang Y.. (2020). A rapid and accurate detection approach for multidrug-resistant tuberculosis based on PCR-ELISA microplate hybridization assay. Lab. Med. 51, 606–613. doi: 10.1093/labmed/lmaa016 32447387

[ref80] Zingue D. Weber P. Soltani F. Raoult D. Drancourt M. (2018). Automatic microscopic detection of mycobacteria in sputum: a proof-of-concept. Sci. Rep. 8:11308. doi: 10.1038/s41598-018-29660-8, PMID: 30054578 PMC6063956

